# Cocaine abuse that presents with acute scrotal pain and mimics testicular torsion

**DOI:** 10.1590/S1677-5538.IBJU.2015.0663

**Published:** 2016

**Authors:** José Tadeu Nunes Tamanini, Vagner Tadeu Salzani, Juliana Milhomem Tamanini, Filipe Iessenco, Leonardo O. Reis

**Affiliations:** 1 Departamento de Medicina da Universidade Federal de São Carlos, SP, Brasil; 2 Faculdade de Medicina ABC, Santo André, SP, Brasil; 3 Departamento de Urologia da Universidade Estadual de Campinas, UNICAMP, SP, Brasil

**Keywords:** Spermatic Cord Torsion, Scrotum, Cocaine

## Abstract

Report case (s) relevant aspects: Man, 27 years old, complaining of acute testicular pain by 2 hours in the remaining left testicle. Denies fever, lower urinary tract symptoms such as dysuria, urinary frequency, concommitant or prior urethral discharge to the painful condition. He underwent right orchiectomy 13 years ago by testicular torsion. He is a chronic user of cocaine for 15 years and during the last three days the drug use was continuous and intense.

Proposed premise substantiating case (s) description:

Initial diagnostic hypothesis:

Syndromic:

Acute Scrotum Syndrome (SEA)

- Main Etiologic (testicular torsion)
- Secondary Etiologic (acute orchiepididymitis)

Briefly delineates what might it add? Lines of research That Could be Addressed: In this challenging clinical case we presented an alternative and new etiologic diangosis for the acute scrotum which the main etiologic factor remains testicular torsion. This new diangosis is acute testicular ischemia as a complication of cocaine abuse.

## INTRODUCTION

Male patient, 27 years-old, got into the emergency room of the local hospital complaining of severe testicular pain that started 2 hours ago and has worsened since then. Moreover, he reported having made use of large amounts of cocaine in the last three days, with the last dose consumed by nasal aspiration within the last 3 hours. He states, as personal history, that underwent orchiectomy 13 years ago due to testicular torsion.

On admission the patient was conscious, alert, oriented and hectic. Pulse=HR=125 beats/minute; Respiratory Rate=22 breaths/minute and blood pressure=150 × 100mm Hg.

**Figure-1:**
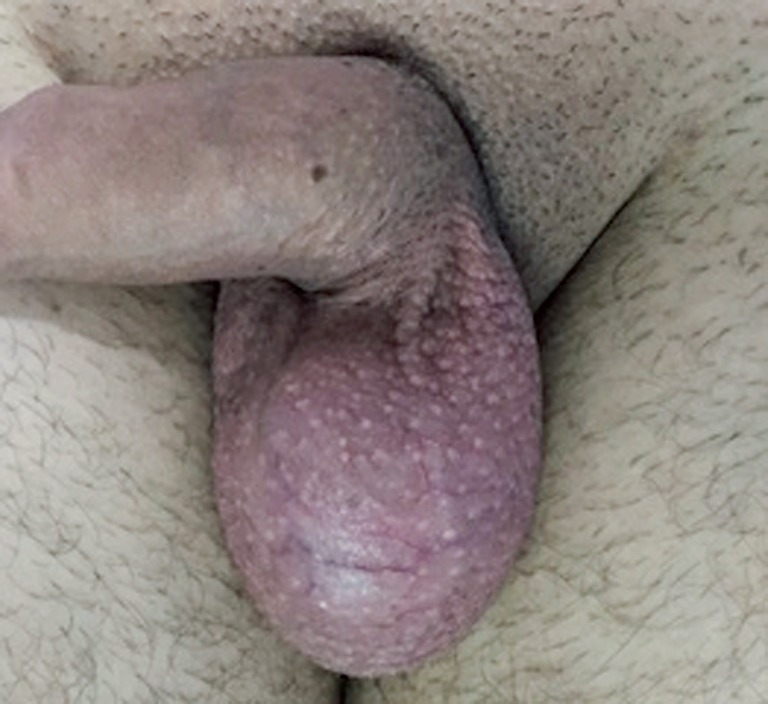
Scrotum with increased volume without wall edema in the preoperative period.

Urological physical examination: penis with normal characters. Scrotal pouch showing remaining left hard testicle, slightly increased in volume and moderate pain on palpation. There were no signs of testicular tumors on palpation, or swelling in the scrotal wall (Figure-1).

After initial approach, the patient was referred immediately to Ultrasonography Unit, whose examination showed reduced blood flow in the left testicle, as well as heterogeneous echo-texture.

With these sonographic findings the most likely diagnosis was “asynchronous testicular torsion”, and the patient immediately was referred for surgical exploration.

**Figure-2:**
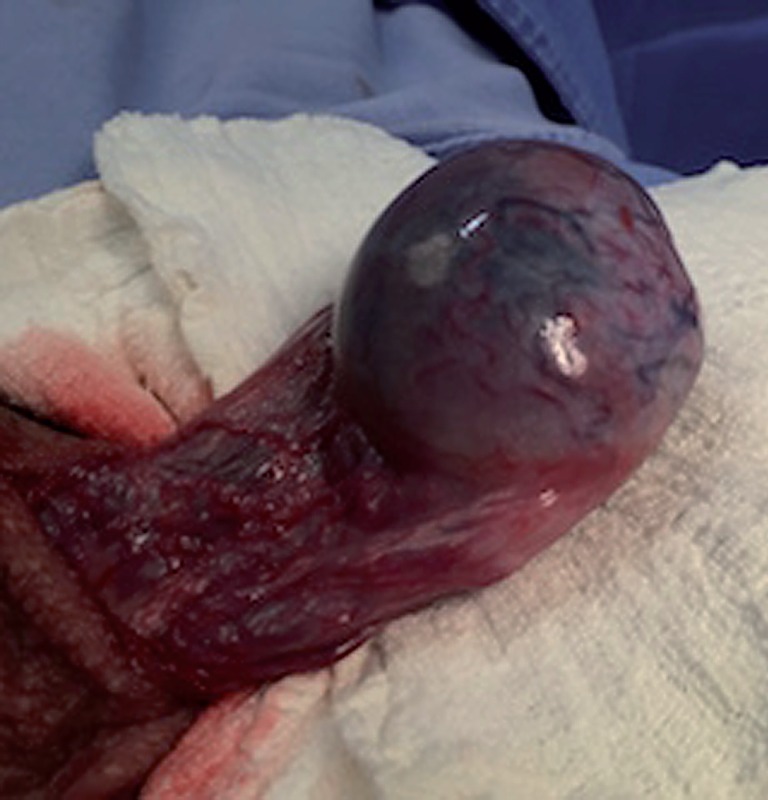
Scrotal exploration showing spermatic cord not twisted and hardened, with areas of ischemia and cyanosis on its surface.

After the left testicle surgical exposure it was noted that there was no testicular torsion or twisting of the spermatic cord, as well as spermatic cord edema. However, the testis was increased in volume and hardened on palpation. The surface was white-pearlescent interspersed with several areas of cyanosis and ischemia, suggesting severe vasoconstriction of the testicular vasculature (Figure-2).

Ventilatory support and hydration was started and urological treatment consisted of immersing the testis in warm saline, followed by surgical fixation, avoiding future testicular torsion.

After 20 minutes of soaking it in warm saline, its surface seemed less cyanotic and having a softer consistency.

Next, scrotal wall was closed in layers with absorbable sutures. Pain improved only after cocaine poisoning has been controlled, about 12 hours later.

Drug testing to identify the poisoning of cocaine was not performed. The diagnosis was eminently clinical, since the clinical history and physical examination confirmed the hypothesis of acute drug intoxication.

### Case (s) hypothesis and rational

**Precepts, clinical and basic reasoning supporting the case (s) and the hypothesis raised scenario. Why is it important and is being Reported?**

After an extensive review of literature and as far as we know, this is the first published case report in the literature related to Acute Scrotum Syndrome caused by ischemia. Furthermore, its main etiology is cocaine abuse, which presents with clinical features that mimic the twist testis or spermatic cord. In fact, it is related to vasoconstriction by chronic cocaine use, worsened with the intensive and acute drug use in the last three days.

It is important to be reported because it can become a differential diagnosis of acute scrotum syndrome. Physicians should consider in the differential diagnosis, since the use of illicit drugs, such as cocaine, is quite prevalent among adolescents and young adults around the world.

**Discussion and future perspectives: what it might add and how does it relate to the current literature**

**‘Take - home message’ - lessons learned**

The sudden onset of clinical symptoms, within a short period of time, scrotal pain or the elements of its contents, with or without increase in scrotal volume is an urologic emergency. The typical presentation is known as Acute Scrotum Syndrome and usually presents in adolescents and young adults. However, some cases are described in newborns (1).

The spectrum of conditions affecting the scrotum and its content ranges from incidental findings to acute pathologic events that require diagnosis and emergency treatment.

The most common differential diagnoses are twisting of the spermatic cord, testicular or appendix torsion, epididymitis, orchitis, inguinal hernia, local trauma, sexual abuse, testicular tumor, scrotal idiopathic edema, cellulitis, vasculitis among others and the ultrasound-Doppler examination is important tool for elucidate the etiologic diagnosis (2).

A careful history and physical examination are of vital importance for the correct diagnosis. In addition, depending on the underlying disease that causes Acute Scrotum Syndrome, time is a critical factor for treatment success.

Testicular Torsion is a condition which must always be considered in cases of acute scrotum syndrome and orchiectomy may be the final result of diagnosis and treatment delay.

This is a 27 year-old man diagnosed with Acute Scrotum syndrome with typical characteristics presented by the acute onset of the history of scrotal pain. The patient had a similar episode of acute scrotum 13 years ago due to a spermatic cord torsion, which resulted in right orchiectomy when he was only 13 years old.

Knowing that the current acute symptom might have the same etiology, we were asked for scrotal ultrasound-doppler, which was made in less than 20 minutes. The sonographic/doppler diagnosis was reduced blood flow to the remaining testicle, strongly suggesting the diagnosis of torsion of the spermatic cord or testis. Thus, the patient was immediately taken to the surgical scrotal exploration (Figure 1 and 2).

From the intraoperative clinical findings it was necessary to return to clinical history to discover the true etiology of the present Acute Scrotum Syndrome.

During the examination of the patient in the emergency room the patient had altered vital signs such as tachycardia, tachypnea and psychomotor agitation, which improved after the use of sedatives in pre-and intraoperative periods.

Testicular pain became more intense when he began to abuse cocaine three hours before seeking medical help. Coincidentally, he said that the pain worsened than ever after cocaine abuse.

The action of cocaine in testicular circulation is very little reported in the literature. In 1999, Li and colleagues reported the effects of cocaine on testicular circulation in rats (3, 4). The ischemic effect is made by indirect action of cocaine by blocking the reuptake of neurotransmitters, which leads to the local vasoconstriction.

Yang and colleagues in 2006 reported that the use of cocaine in rats resulted in prolonged vasoconstriction in testicular vessels and the effects of cocaine in the testis, as infertility, may be due to ischemia or ischemia-reperfusion injury of the testis (5).

In humans, the relationship between testicular pain and ischemia induced by chronic use of cocaine was initially suggested by Neynaber and colleagues in a case report of Wegener’s granulomatosis. However, testicular pain in this case was part of the clinical syndrome, where other organs were affected simultaneously. It was described a case of a young man (22 years old-cocaine addict) who developed vasculitis with involvement of multiple organs such as the upper and lower respiratory tract, kidney, testicles and skin as well as laboratory abnormalities. All these characteristics lead to a diagnosis of Wegener’s granulomatosis. The involvement of multiple organs is typical of this syndrome; however, testicular involvement is very rare; it was believed that cocaine abuse had stimulated the development of systemic granulomatous vasculitis (6).

Doherty and colleagues reported the patient’s history with acute testicular pain that mimicked the clinical condition of torsion of the spermatic cord.

Ultrasound examination revealed impaired testicular blood flow compatible with the main hypothesis and the patient underwent emergency scrotal exploration. However, it was not found torsion of the spermatic cord. Reviewing the clinical history, the authors observed that the patient was daily user of metanphetamin and had used the drug one hour before starting pain in the acute scrotum syndrome (7). The description of this event has the same context of the present case report, however with different drug use (8).

In a study that evaluated the anatomical aspects of the epididymis and tunica vaginalis in patients with testicular torsion, Favorite and colleagues (9) found that the normal anatomy of the contralateral epididymis and tunica vaginalis was rare (4% in their series). According to these authors, these findings highlight the need for exploration and bilateral orchipexy in cases of testicular torsion.

In this case, although we found no evidence of testicular torsion in the surgical approach, we proceeded the fixation as a protective procedure justified by his past history of testicular torsion.

As differential diagnosis we remember that Polyarteritis nodosa (PAN) is a vasculitis of immune origin that can cause clinical involvement of testicular pain in 2–18% of cases. Testicular pain is one of 10 criteria to make the diagnosis of PAN according to the American College of Rheumatology (10).

This is the first case described in the literature that presents with clinical features that mimic testicular torsion of the spermatic cord or without other systemic symptoms. Unlike other cases in the literature, this patient had testicular pain as the only symptom of acute scrotum syndrome.

The effects of the use and abuse of cocaine are well described in the literature. Patients may present tachycardia, hypertension, and psychomotor agitation. Concerning Central Nervous System symptoms, patients may have seizures, ischemic or hemorrhagic stroke and its clinical consequences. In the cardiovascular system, they may present hypertension and tachycardia, cardiac arrhythmias, myocardial infarction even in young patients without atherosclerosis. The respiratory system may have chronic rhinitis, nasal septum perforation, oropharyngeal ulcers and pulmonary infarction. In the digestive system one can find ulcerations and bowel infarction and ischemic colitis as well. The changes in liver, kidney and skin are also predominantly related to vasoconstriction.

The final message this case report teaches us is that we must consider testicular isquemia as a differential diagnosis of acute scrotum syndrome in patients under cocaine intoxication.
